# The Orexin/Receptor System: Molecular Mechanism and Therapeutic Potential for Neurological Diseases

**DOI:** 10.3389/fnmol.2018.00220

**Published:** 2018-06-28

**Authors:** Chunmei Wang, Qinqin Wang, Bingyuan Ji, Yanyou Pan, Chao Xu, Baohua Cheng, Bo Bai, Jing Chen

**Affiliations:** ^1^Neurobiology Key Laboratory of Jining Medical University in Colleges of Shandong, Jining Medical University, Jining, China; ^2^Division of Biomedical Sciences, Warwick Medical School, University of Warwick, Coventry, United Kingdom

**Keywords:** orexin, receptor, neuroprotective, pathway, neurological disease, molecular mechanisms

## Abstract

Orexins, also known as hypocretins, are two neuropeptides secreted from orexin-containing neurons, mainly in the lateral hypothalamus (LH). Orexins orchestrate their effects by binding and activating two G-protein–coupled receptors (GPCRs), orexin receptor type 1 (OX1R) and type 2 (OX2R). Orexin/receptor pathways play vital regulatory roles in many physiological processes, especially feeding behavior, sleep–wake rhythm, reward and addiction and energy balance. Furthermore several reports showed that orexin/receptor pathways are involved in pathological processes of neurological diseases such as narcolepsy, depression, ischemic stroke, drug addiction and Alzheimer’s disease (AD). This review article summarizes the expression patterns, physiological functions and potential molecular mechanisms of the orexin/receptor system in neurological diseases, providing an overall framework for considering these pathways from the standpoints of basic research and clinical treatment of neurological diseases.

## Introduction

The orexins, also known as hypocretins, are a pair of neuropeptides that are mainly derived from orexin-containing neurons in the lateral hypothalamus (LH). Orexin-A (OA; hypocretin-1) and orexin-B (OB; hypocretin-2) are closely related small peptides that are widely distributed throughout the central and peripheral nervous systems (de Lecea et al., 1998; Sakurai et al., 1998). Orexins stimulate food intake upon intracerebroventricular administration, and were originally described as regulators of feeding behavior (Yamanaka et al., 1999). Subsequent experiments revealed many other important physiological functions of these peptides, including regulation of the sleep–wake cycle (de Lecea and Sutcliffe, 2005; Chow and Cao, 2016), energy homeostasis (Tsuneki et al., 2012), neuroendocrine functions (Inutsuka and Yamanaka, 2013), glucose metabolism (Tsuneki et al., 2016), stress-adaptive responses (Xiao et al., 2013) and reward-seeking and drug addiction (Aston-Jones et al., 2010).

Orexins bind their cognate G-protein–coupled receptors (GPCRs), orexin receptor type 1 (OX1R, also named as Hcrtr-1) and type 2 (OX2R, or Hcrtr-2), which activate different downstream signal pathways, thereby exerting a variety of physiological functions (Sakurai et al., 1998). Orexin and orexin receptors are ectopically expressed in many diseases (Perez et al., 2015; Imperatore et al., 2017), especially neurological disorders (Feng et al., 2014; Liguori et al., 2014), suggesting that the orexin/receptor pathway plays critical roles in the pathology and pathogenesis of these illnesses. In this review article, we focus on the expression levels and physiological functions of orexins and their receptors. In addition, we discuss the potential contributions of the orexin/receptor pathway in neurological diseases such as narcolepsy, drug addiction, depression, ischemic stroke, and Alzheimer’s disease (AD). Together, these discussions summarize our current knowledge of the orexin/receptor system and the prospects for applying this information to the clinical treatment of neurological diseases.

## Orexin Structure

In 1998, two experimental groups nearly simultaneously discovered a pair of new neuropeptides in the LH and adjacent areas of rat brain. One group (de Lecea et al., 1998) used DNA subtractive hybridization, whereas the other used a high-performance liquid chromatography (HPLC) approach (de Lecea et al., 1998; Sakurai et al., 1998). de Lecea et al. (1998) named the peptides hypocretin-1 and hypocretin-2, respectively, because they are expressed specifically in the posterior hypothalamus. On the other hand, Sakurai et al. (1998) called them OA and OB after the Greek word for “appetite”.

**Figure 1:**
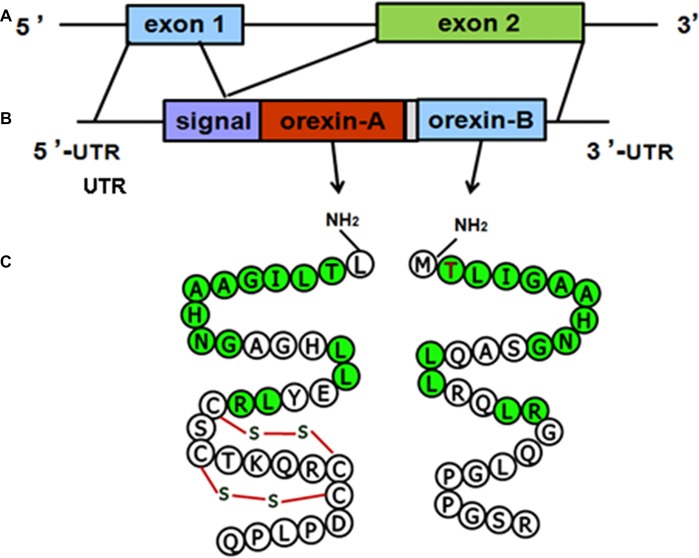
Molecular structures of orexin precursor and orexins. **(A)** Genomic DNA of human prepro-orexin containing two exons and one intron. **(B)** The mRNA of human orexin, including the 5’ untranslated region (UTR), signal peptide, open reading frame (ORF) encoding orexin-A (OA) or orexin-B (OB), and 3’ UTR. **(C)** Amino acid sequences of OA (33 aa) and OB (28 aa). Green labels indicate amino acids that are identical in both OA and OB.

The structure of the orexin gene, which consists of two exons and one intron, is conserved in all vertebrates. The genomic DNA sequence encoding human orexin contains 1432 base pairs (bp). The first exon includes the 5’ untranslated region (UTR) and the first seven amino acids (aa) of the signal peptide, and the second exon contains the remainder of the open reading frame (ORF) and the 3’ UTR. The mRNA sequences of human orexin contain 616 bases, encoding a precursor peptide (prepro-orexin) that contains 131 aa residues. The first 33 aa of prepro-orexin constitute the signal peptide, which is followed by 33 and 28 aa in OA and OB, respectively, which share 46% (13/28) amino acid identity (Sakurai et al., 1998; Alvarez and Sutcliffe, 2002). The molecular structures of the orexin precursor and orexin are shown in (Figure 1).

**Figure 2:**
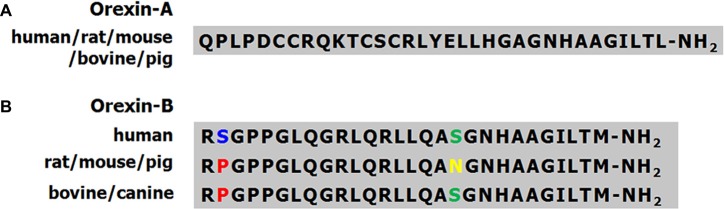
Amino acid sequences of OA and OB in different species. **(A)** The amino acid sequences of OA are identical in human, rat, mouse, bovine and pig. **(B)** The amino acid sequences of OB are highly conserved in human, rat, mouse, bovine and dog. Different colors indicate amino acids of OB that differ among species.

The molecular masses of OA and OB are 3562 Dopamine (DA) and 2937 Da, respectively. OA contains four Cys residues that form two intrachain disulfide bonds (Figure 1C). The OA sequences of human, mouse, rat and cow are identical, and the high level of conservation predicts that OA has important physiological functions (Wong et al., 2011). The mouse and rat OB sequences are also identical, whereas human OB differs from the rodent peptide at two positions (Figure 2). A serine residue at the second position in the human peptide is replaced by a proline in cow and dog. In addition, the serine at the 18th position is replaced by asparagine in rat, mouse and pig.

## Orexin Receptors

OA and OB orchestrate their diverse effects by binding and activating two GPCRs, OX1R and OX2R. The mRNAs of human OX1R and OX2R are 1564 and 1843 bp in length, respectively, and are translated into proteins of 425 and 444 aa. OX1R and OX2R share 64% amino acid identity. Rat OX1R and OX2R mRNAs are 2469 and 3114 bp in length, respectively, and encode proteins of 413 and 460 aa. Human and rat OX1R and OX2R share 94% and 95% identity, indicating that the orexin receptor is highly conserved among mammals.

The original reports describing the orexin receptors revealed that OA and OB bind the receptor subtypes with different affinities (Voisin et al., 2003). OA preferentially binds OX1R, with 5–100-fold greater affinity than OB, whereas both OA and OB have similar affinities for OX2R, a less selective receptor (Sakurai et al., 1998; Ammoun et al., 2003).

Although orexin-containing neurons project widely to various brain regions, the two receptors are distributed differently (Marcus et al., 2001). Both proteins are co-expressed at least in some areas of the central nervous system (Trivedi et al., 1998; Hervieu et al., 2001; Cluderay et al., 2002), such as the ventral tegmental area (VTA), pedunculopontine tegmental nucleus (PPT), and laterodorsal tegmental nucleus (LDT). OX1R is preferentially expressed in the locus ceruleus (LC), whereas OX2R is mainly distributed in the tuberomammillary nucleus (TMN). The selectivity of orexins for OXRs and the distinct distributions of the receptors may be responsible for the differential physiological effects of the orexin/receptor pathway.

## Orexin/Receptor Signaling Pathway

The existence of two orexins and two receptors subtypes is bound to create diversity within cellular signaling pathways. The orexin/receptor signaling pathway has been described in recombinant cell lines and native systems. As in most GPCR-mediated pathways, orexins first bind OXRs, which in turn activate at least three subtypes of G-proteins (G_q/11_, G_i/o_, and G_s_) or other proteins (e.g., β-arrestin). These effectors subsequently regulate phospholipases, ion channels, and protein kinases, ultimately triggering the activation of various downstream signaling pathways (Dalrymple et al., 2011; Kukkonen and Leonard, 2014; Leonard and Kukkonen, 2014).

Calcium is a very important second messenger of GPCR-mediated signaling. Previous studies demonstrated that orexin treatment significantly increases the intracellular Ca^2+^ concentration ([Ca^2+^]_i_) in cells overexpressing OX1R and OX2R, an effect that is mainly triggered by activation of the classical phospholipase C (PLC) cascade (PLC-IP_3_/DAG; Lund et al., 2000; Ammoun et al., 2006a; Johansson et al., 2008). Subsequent work showed that OA acts on OX1R, which in turn activates transient receptor potential channel 3 (TRPC3), thereby triggering Ca^2+^ responses (Peltonen et al., 2009). These results revealed that orexin receptors activate a novel mechanism of [Ca^2+^]_i_ elevation via nonselective cation channels (NSCCs), in contrast to the original notion that the changes in calcium levels were mediated primarily through GPCRs.

**Figure 3:**
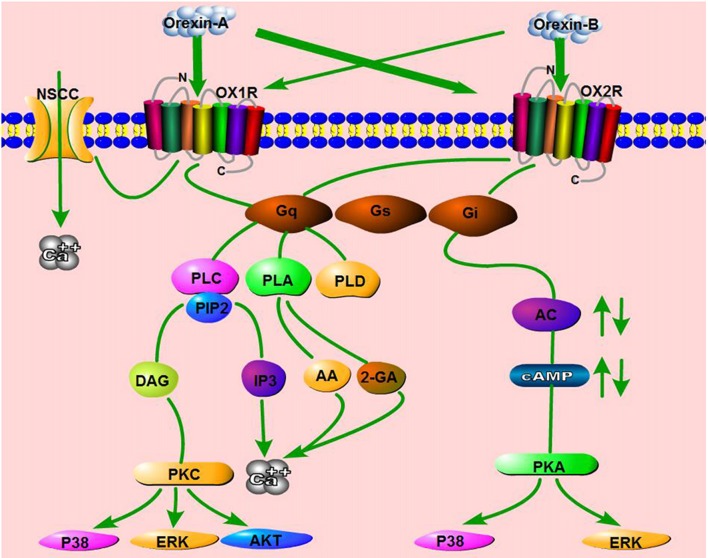
Schematic summary of signaling pathways mediated by the orexin/receptor system. The binding of orexins to orexin receptor type 1 (OX1R) or OX2R stimulates G_q_ or G_i_ subtypes, which subsequently induce the activation of phospholipase C (PLC), phospholipase A (PLA), phospholipase D (PLD) or Adenylyl cyclases (AC), ultimately resulting in an increase in cytosolic Ca^2+^ and a downstream cascade response. In addition, OA binds OX1R and elevates Ca^2+^ by activating nonselective cation channels (NSCCs).

Other orexin/receptor signaling pathways have also been reported, including the phospholipase D (PLD)/phosphatidic acid (PA; Johansson et al., 2008), phospholipase A (PLA)/arachidonic acid (AA; Turunen et al., 2012), and mitogen-activated protein kinase (MAPK) cascades (Ramanjaneya et al., 2009). Human OX1R potently activates PLD1 via nPKC, but not Rho-family G-proteins, in CHO cells stably expressing human OX1R (Jäntti et al., 2012). In HEK293 cells expressing OX1R, as well as in Neuro-2a cells, stimulation of OX1R by OA liberates both 2-arachidonoyl glycerol (2-AG) and AA (Turunen et al., 2012). Orexins also activate p38-MAPK signaling pathway and increase the level of phosphorylated ERK1/2 in a dose- and time-dependent manner in both cell lines and tissues (Milasta et al., 2005; Ammoun et al., 2006b). ERK_1/2_ activation induced by orexins involves Gq/PLC/PKC signaling, but not the PKA pathway (Wenzel et al., 2009). Ramanjaneya et al. also showed that ERK_1/2_ and p38 are phosphorylated rapidly in response to OA and OB, predominantly mediated by G_q_ and, to a lesser extent, G_i_ (Ramanjaneya et al., 2009). In addition, OA affects rat insulinoma cell proliferation via stimulation of the AKT signaling pathway by OX1R (Chen et al., 2013). These results show that the orexin/receptor system can activate potent intracellular signaling via pathways other than the classical signaling pathways (Figure 3).

In neuron-like cells, orexin/receptor pathways are similar to those described above, in which activation of PLC and [Ca^2+^]_i_ elevation play central roles in signaling (Holmqvist et al., 2002). In various types of neurons, orexins bind OXRs and subsequently activate intracellular calcium signaling through PKC-dependent or -independent pathways (Ozcan et al., 2010). For example, OA elevates [Ca^2+^]_i_ by activating L- and N-type Ca^2+^ channels, an effect mediated by phosphatidylcholine-specific PLC and PKC in dopaminergic neurons (Uramura et al., 2001). The combination of OA and OX1R induces an increase of [Ca^2+^]_i_ in prefrontal cortex neurons, dependent upon extracellular Ca^2+^ influx via L-type Ca^2+^ channels activated by the intracellular PLC–PKC signaling pathway (Xia et al., 2009). Through OX1R–PLC–PKC, OA upregulates the voltage-gated L-type calcium channel, which subsequently activates the hypothalamic AMPK signaling pathway in NPY neurons (Wu et al., 2013). Orexins also increase postsynaptic [Ca^2+^]_i_ via OX2R, and the increase in [Ca^2+^]_i_ is induced by the AC-PKA–mediated activation of voltage-gated R- and T-type Ca^2+^ channels (Nakamura et al., 2010). Orexins stimulated Akt kinase activation in rat cortical neurons subjected to hypoxic stress, and the pro-survival action of orexins displayed in a concentration- and time-dependent manner (Sokoowska et al., 2014). In addition, in differentiated neuroblastoma cells, OA–linked OX1R increases the influx of Ca^2+^ through diacylglycerol-activated channels, which are inhibited by activated PKC (Nasman et al., 2006).

Coupling between orexin/receptor and Adenylyl cyclases (AC)/cAMP has been reported in several studies. For example, in cultured rat cortical neurons, OA and OB stimulation causes OX2R to couple to G_i_ proteins, leading to inhibition of cAMP formation (Urbanska et al., 2012). However, OA also activates OX1R to stimulate cAMP synthesis in cultured rat astrocytes (Woldan-Tambor et al., 2011). Furthermore, orexin/receptor signaling rapidly activates the mTORC1 pathway, which is triggered by the lysosomal v-ATPase pathway, which is in turn dependent on transient cytoplasmic calcium (Wang Z. et al., 2014).

Generally speaking, OA/OX1R pathways have been examined in much greater detail than orexin/OX2R pathways, although recent studies have begun to elucidate the latter. In general, the responses to OX2R activation by orexins are similar to those to OX1R. However, some of the activations are weaker, indicating a differential coupling of the two receptors to certain cascades. Finally, a more detailed network analysis is essential to elucidate the molecular mechanisms underlying the intracellular effects of the orexin/receptor system, as well as to develop new clinical approaches to treat diseases related to this pathway.

## Dimerization of Orexin Receptors

**Figure 4:**
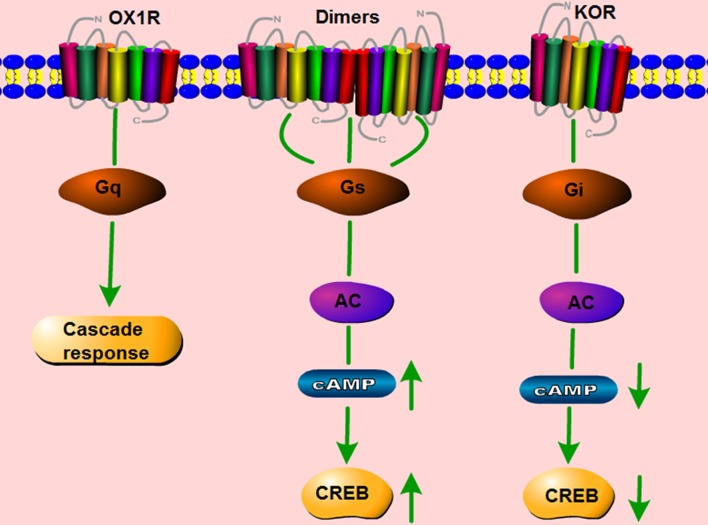
Schematic change in signaling pathway mediated by OX1R–kappa opioid receptor (KOR) heterodimers. OX1R mainly activates the G_q_ subtype, which subsequently induces a downstream cascade response. KOR binds the G_i_ subtype, which inhibits the activity of cAMP and p-CREB. The dimerization of OX1R and KOR changes the primary signaling pathway, but activates G_αs_ subtypes, which increase the activity of cAMP and p-CREB.

It is traditionally accepted that GPCRs exist and function as monomers. However, a large number of studies show that GPCRs can also form homo- and heterodimers, and that these dimers have distinct effects on the signaling pathways induced by the corresponding monomers (Bulenger et al., 2005; Cottet et al., 2010). OX1R and the CB1 receptor are present as heterodimers/oligomers *in vitro* (Ellis et al., 2006). Moreover, OX1R and OX2R easily form homo- and heteromeric complexes with each other. CB1 receptors form homodimers, and they also form heterodimers with both orexin receptors (Jäntti et al., 2014). Our lab studied whether two GPCRs co-expressed in the same cerebral area can form dimers, and if so, whether such dimerization is involved in the pathology of neurological disorders. In HEK293 cells co-transfected with mouse orexin receptors mOX2αR and mOX2βR, we observed dimerization between mOX2αR and mOX2βR using bioluminescence resonance energy transfer (BRET) and co-immunoprecipitation (Co-IP; Wang C. et al., 2014). Dimerization of mOX2αR and mOX2βR causes a greater increase in p-ERK_1/2_ and intracellular Ca^2+^ elevation after stimulation with OA or OB than occurs in cells transfected with mOX2αR or mOX2βR alone. In addition, using fluorescence resonance energy transfer (FRET), we showed that both OX1R and kappa opioid receptor (KOR) can heterodimerize. The heterodimer binds Gα proteins, leading to activation of the PKA signaling pathway, including upregulation of cAMP levels and the cAMP-response element (CRE; Figure 4; Chen et al., 2015). We also observed that OX1R and CCK1R heterodimers inhibit the activation of G_αq_, G_αi2_, G_α12_, and G_α13_ in comparison with stimulation by OA or CCK alone. In these experiments, endogenous OX1R and CCR1R were expressed in HT-29 cells using Duolink II *in situ* PLA detection kits. Moreover, OX1R/CCK1R heterodimers affected the migration of HT-29 cells, suggesting that OX1R/CCK1R heterodimerization plays an anti-migratory role in human colon cancer cells (Bai et al., 2017). All of these results indicate that the heterodimers of different GPCRs can perform specific functions distinct from those of the constituent monomers, leading to various effects on physiological processes.

## Orexin/Receptor Signaling and Neurological Diseases

Although orexin-containing neurons are distributed only in the LH and adjacent areas, their nerve fibers project widely to multiple brain regions (Chen et al., 1999; Nambu et al., 1999). Furthermore, orexin receptors exhibit a diffuse distribution corresponding to this widespread projection (Marcus et al., 2001). The extensive projection of orexin innervation and the characteristic expression of receptors appear to provide the structural basis by which the orexin/receptor system participates in regulation of multifaceted functions. Current studies support the idea that the orexin/receptor system is involved in controlling the activity of the nervous system. In particular, OA can rapidly cross the blood–brain barrier by simple diffusion (Kastin and Akerstrom, 1999). Furthermore, the prepro-orexin gene is located on chromosome 17q21, making it a candidate gene for neurological disorders (Wilhelmsen, 1997). Indeed, accumulating evidence shows that the orexin/receptor system is ectopically expressed in several neurological disorders, suggesting that it plays an important role in the incidence and pathogenesis of these diseases.

### Orexin/Receptor Signaling and Narcolepsy

Narcolepsy is a chronic sleep disorder characterized by cataplexy, sleep paralysis, excessive sleep, hypnagogic hallucinations and abnormal transition from wakefulness to rapid eye movement (REM) sleep (Mignot, 1998). It is widely believed that narcolepsy is closely related to disorders of the hypothalamus and abnormalities of the histamine system (John et al., 2004). The important role of the orexin/receptor system in both of the above strongly suggests that it is involved in the pathogenesis of narcolepsy.

Multiple studies report that deficiencies in the orexin/receptor system are associated with human narcolepsy. Consistent with this, disruption of the system causes narcoleptic symptoms in animal models. Lin et al. (1999) first cloned the mutant *OX2R* gene in a canine narcolepsy model and showed that disruption of *OX2R* caused canine narcolepsy, suggesting a therapeutic target for the treatment of narcoleptic patients. Simultaneously, mice harboring a knockout of the prepro-orexin gene manifest a narcolepsy-like phenotype, remarkably similar to human and canine narcolepsy, suggesting that narcolepsy is associated with the orexin system (Chemelli et al., 1999). Early in 1999, Nishino and co-workers found that the OA concentration in cerebrospinal fluid (CSF) was abnormally low in seven of nine people with narcolepsy, implying that orexin transmission was deficient in these patients (Nishino et al., 2000). In a later study, the same group reported a dramatic decrease in the CSF OA levels in 32 of 38 successive narcolepsy–cataplexy cases (Nishino et al., 2001b). On the basis of these findings, they concluded that orexin is deficient in most cases of human narcolepsy, suggesting possible diagnostic applications. Furthermore, the number of orexin neurons is reduced by 85%–95% in the LH of patients with narcolepsy (Thannickal et al., 2000). Orexin mRNA and neuropeptide are completely absent in hypothalamus, pons and cortex of narcolepsy patients, and the secretory signal sequence of the orexin gene is deficient in the most serious cases of early onset narcolepsy (Peyron et al., 2000). These observations further prove that narcolepsy is associated with deficiency in the orexin system.

Although OX1R has a mild effect on sleep–wake behavior, only OX2R- and OX1R/OX2R-knockout mice exhibit narcoleptic symptoms, with more severe phenotypes in the double knockout (Beuckmann et al., 2004; Scammell and Winrow, 2011). Additionally, OX2R and dual orexin receptor antagonists, but not OX1R antagonists, inhibit wakefulness (Kalogiannis et al., 2011). These results showed that narcoleptic effects are mainly mediated by OX2R or a combination of OX1R and OX2R, but not by OX1R alone.

Orexin-containing neurons not only innervate target neurons via efferent nerves, but also accept projections from their target neurons, particularly monoaminergic (i.e., noradrenergic; Sakurai, 2007), serotonergic (Brown et al., 2001), histaminergic (Eriksson et al., 2001), dopaminergic (Korotkova et al., 2003), and cholinergic neurons (Burlet et al., 2002). Orexin and monoaminergic neurons form a negative feedback pathway in the dorsal raphe nucleus (DR) and LC (Brown et al., 2002; Muraki et al., 2004). Thus, activity of orexin neurons may increase the activity of monoaminergic neurons. Conversely, monoaminergic neurons may decrease the inhibitory effect on orexin neurons, thereby stabilizing wakefulness through this dynamic mechanism. Moreover, two efferent pathways of orexin-containing neurons are likely to be mediated by DR serotonergic and LC noradrenergic neurons. Restored expression of OXR in the DR and LC of mice lacking OXR inhibits cataplexy-like episodes, and the degree of suppression correlates with the number of serotonergic neurons in the DR in which OXR expression is restored, whereas maintenance of wakefulness correlates with the number of noradrenergic neurons restored in the LC (Hasegawa et al., 2014). In addition, OA/receptor signaling in DR serotonergic neurons that express both OX1R and OX2R plays a pivotal role in the prevention of cataplexy-like episodes (Hasegawa et al., 2014). Indeed, DA levels are high in several brain structures of narcoleptic Dobermans and postmortem brain of humans with narcolepsy (Nishino and Mignot, 1997; Nishino et al., 2001a), consistent with the hypothesis that altered DA accounts for the sleep abnormalities in hypocretin-deficient narcolepsy. Using orexin-knockout mice as a model of human narcolepsy, DA was shown to suppress cataplexy mediated by D2-like receptors and sleep attacks modulated by a D1-like receptor, confirming that dopaminergic mechanisms contribute to narcolepsy symptoms (Burgess et al., 2010).

**Figure 5:**
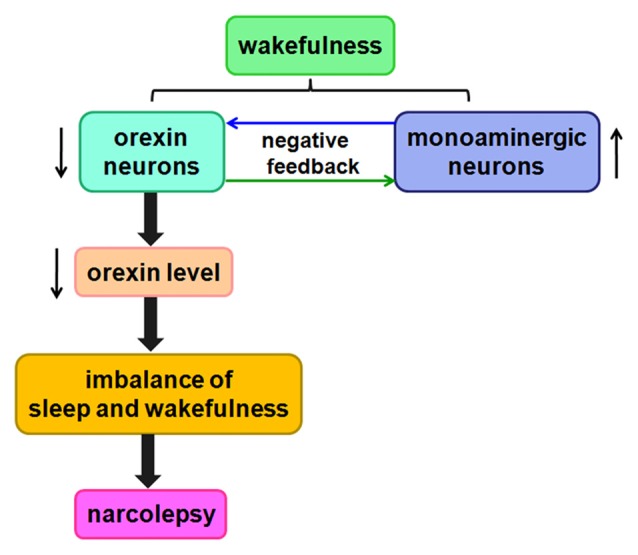
Likely relationship between orexin and narcolepsy. A negative feedback pathway connects orexin neurons and monoaminergic neurons. When orexin neurons are damaged, orexin levels are reduced, causing an imbalance among these neurotransmitter systems, likely leading to narcolepsy.

These results show that the orexin/receptor system probably promotes wakefulness and inhibits sleep by regulating complex circuits. Orexin-containing neurons gradually die over the course of narcolepsy, thereby decreasing orexin levels. Ultimately, this process contributes to imbalances among these neurotransmitter systems, likely giving rise to the symptoms of narcolepsy (Figure 5).

### Orexin/Receptor Signaling and Insomnia

Insomnia is a chronic and pervasive sleep disorder in the world. Insomnia is characterized by difficulty initiating asleep and/or staying asleep that easily leads to impairment of daytime functioning, such as daytime sleepiness, fatigue, irritability, memory impairment and other symptoms (Roth and Roehrs, 2003; Ishak et al., 2012). Currently, treatment for insomnia in clinical is cognitive behavioral therapy combined with pharmacological therapy (Holbrook et al., 2001; Equihua et al., 2013). However, pharmacological therapy has potential negative effects, such as daytime drowsiness, tolerance, dependance and withdrawal symptoms (Dundar et al., 2004; Lieberman, 2007), thus new insomnia therapy with less negative effects is under investigation.

The orexin/OXR system strongly involves in regulating the sleep-wake cycle owing to its role in promoting and sustaining wakefulness (Piper et al., 2000). In experiment of freely moving cats, OA levels in day were higher during active waking than during slow-wave sleep (SWS). Moreover, OA levels were significantly higher during REM sleep than during SWS (Kiyashchenko et al., 2002). In a experiment using squirrel monkeys, OA levels in CSF were lower on awakening and gradually increased over the course of the day, coinciding with greater levels of activity; but its levels decreased during the night when they were asleep (Zeitzer et al., 2003). These results suggested that orexin levels have a significant correlation to the time of day. On the contrary, overexpression of the orexin/OXR system is bound to disrupt the sleep-wake cycle. In an experiment using zebrafish, insomnia-like phenotype had been shown after orexinergic neurons were overexpressed (Prober et al., 2006). OA levels had significantly higher in the patients with insomnia disorder than normal sleepers. Moverover, OA levels were detected to have a positive relationship with the course and severity of insomnia (Tang et al., 2017).

Because the orexin/OXR system promotes wakefulness, antagonists that block orexin receptors would promote sleep and inhibit wakefulness. This must offer an important and effective therapeutic alternative for insomnia. Some orexin receptor antagonists have been designed and researched for the treatment of insomnia in rats, dogs and humans (Brisbare-Roch et al., 2007), including two categories: single orexin receptor antagonists (SORAs) and dual orexin receptor antagonists (DORAs).

SB-334867, one of SORAs, was the first drug designed to selectively antagonize OX1R (Smart et al., 2001). It may counteract the suppression of REM sleep in rats, but it may not decrease wakefulness, or increase the amount of time spent in sleep (Smith et al., 2003). OX2R antagonists, such as EMPA, TCS-OX2–29 and JNJ-10397049, have been more effective on diminishing wakefulness than OX1R antagonists.

DORAs that are currently widely discussed are SB-649868, almorexant and suvorexant. In preclinical studies, administration of SB-649868 attenuated grooming activity induced by OA in rats. Moreover, SB-649868 (3–30 mg/kg) significantly reduced latency and increased the duration of non-REM and REM sleep (Di Fabio et al., 2011). Furthermore, phase I polysomnography data indicated that SB-649868 significantly shortened time to persistent sleep and obviously improved total sleep time (Bettica et al., 2012). Almorexant has been proved to increase REM and non-REM sleep in a dose-dependent manner, and decrease locomotion induced by OA in mice and rats (Mang et al., 2012). Suvorexant significantly reduced wakefulness and increased REM and NREM sleep in rats, dogs and rhesus monkeys in a dose-dependent manner (Whitman et al., 2009; Winrow et al., 2011). A crossover trial of suvorexant showed that it significantly improved the sleep efficiency of insomnia patients (Herring et al., 2012). Because of the efficacy and tolerability in Phase II and III trials of suvorexant, the FDA in the 2014 approved suvorexant as a first-in-class DORAs for the treatment of insomnia (Winrow and Renger, 2014).

In fact, insomnia treatments are usually complex and difficult in clinical becuase insomnia is a multifactorial etiology. The design and availability of orexin receptor antagonists as an effective alternative would be an important development in insomnia management. However, the efficacy of antagonists on the quality and quantity of sleep is not fully understood.

### Orexin/Receptor Signaling and Depression

Depression is a mental illness whose incidence is increasing around the world. The characteristics of depression include low mood, misery, apathy, low self-esteem, anhedonia, loss of motivation, loss of appetite, sleep disorders, retardation of thought and action (Mathers and Loncar, 2006; Ionescu and Papakostas, 2017); consequently, this disease seriously endangers human physical and mental health. Orexin-containing neurons project neurofibers to the dopaminergic ventral tegmental nucleus and substantia nigra, which are important regulators of emotional activity, suggesting that the orexin/receptor system is involved in the pathophysiology of depression.

Dysregulation of the orexin/receptor system has been reported in patients with depression, as well as in animal models of the disease. In 2003, Ronald M. Salomon measured the OA concentration in CSF of patients with depression (Salomon et al., 2003), and showed that the OA level was higher in patients than controls. In addition, the OA level tended to decrease after administration of the antidepressant sertraline for 5 weeks, confirming the correlation between depression and OA. Feng et al. (2008) reported a significant decrease in the level of orexins in a rat model of depression at younger ages, but significantly higher levels of orexins in adult rats, possibly due to disinhibition from defective aminergic neurons. In a rat model of depression, the reduced number and size of orexin neurons are associated with depressive symptoms (Allard et al., 2004). Consistent with this, OA mRNA can be detected in peripheral blood cells of patients with depression on the 1st, 14th and 28th day after admission (Rotter et al., 2011). Together, these results show that OA mRNA levels are negatively correlated with the severity of depression. Additionally, Ito et al. (2008) examined in mice effects of OA given i.c.v. on the forced swim test, an accepted behavioral screen of antidepressant activity. The result suggested that OA induced an antidepressive-like effect, at least partly by the enhancement of cell proliferation in the dentate gyrus (Ito et al., 2008).

However, experiments performed in various animal models of depression yielded contradictory results, as described below. The abundance of orexin-containing neurons in the LH increases by about 20% in depressed mice subjected to high and prolonged external stress, in comparison with age-matched controls (Jalewa et al., 2014). Orexin-containing neurons are also activated in a rodent model of depression, and the increase in the number of neurons is reversed by the antidepressant fluoxetine (Nollet et al., 2011). Expression of orexin in depressive mice varies among brain regions (Arendt et al., 2013). In the hippocampus, lower expression of orexin is negatively correlated with depressive behavior, whereas in the amygdala, higher expression of orexin and *OX1R* mRNA is positively correlated with depressive behavior. These results provide further confirmation that the orexin/receptor pathway plays distinct roles in different brain regions.

OX2R has antidepressive properties, whereas OX1R is pro-depressive. Treatment of a rat model of seasonal affective disorder (SAD) with SB334867, a selective antagonist of OX1R, leads to depressive behaviors characterized by a decrease in sweet solution preference and increased immobility in the forced swim test (Deats et al., 2014). On the other hand, both OX1R-knockout and normal mice treated with SB334867 exhibit similar reductions in behavioral despair, whereas OX2R-knockout mice exhibit an increase in behavioral despair. The authors inferred that the orexin/receptor system induced an antidepressant or pro-depressant effect depending on whether OX1R or OX2R was activated (Scott et al., 2011). OX1R expression differs very little between a mouse model of depression and control mice. By contrast, in the depressed mice, the OX2R level is reduced in the hypothalamus, ventral thalamus and hippocampus (Nollet et al., 2011).

**Figure 6:**
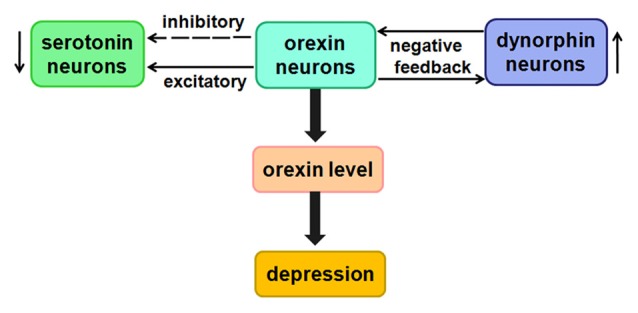
Likely relationship between orexin and depression. Low concentrations of orexins have a direct excitatory effect on 5-HT neurons, whereas higher concentrations of orexins indirectly inhibit 5-HT neurons.

In our lab, we observed that OX1R and KOR are co-expressed in primary rat hippocampal neurons. Notably, both OX1R and KOR are expressed at lower levels in hippocampus and hypothalamus in a mouse depression model than in control mice (Figure 6). However, in comparison with the slight decrease in KOR expression, we observed a far greater decrease in OX1R expression in the depression model. These results suggested that depression may cause an imbalance between OX1R and KOR in brain tissue, consistent with the view that dysregulation of OX1R and KOR promotes the development of depression (Chen et al., 2015). In addition, dynorphin is highly co-localized with orexin in the hypothalamus. In an animal model of depression, the dynorphin level is elevated, whereas the orexin level is reduced, causing symptoms lacking pleasure. Dynorphin and orexin perform reciprocal, antagonistic roles in the regulation of the brain reward system, and are jointly involved in modulating the physiological and pathological processes of depression (Miczek et al., 2011).

The studies showed that orexins may directly excite serotonin (5-HT) neurons by activating K^+^ leak currents or Na^+^-dependent NSCCs (Brown et al., 2001). Unexpectedly, at higher concentrations, orexins indirectly inhibit 5-HT neurons by exciting GABAergic interneurons (Liu et al., 2002). The speculation from current study is that there may be certain relationship between them in the process of depression.

Taken together, this evidence clarifies the important role of the orexin/receptor system in regulating the pathology of depression. These findings not only improve our understanding of the function of the orexin/receptor system, but also provide new insights into the molecular mechanisms of depression.

### Orexin/Receptor Signaling and Ischemic Stroke

Ischemic stroke, a nervous system disease associated with a high mortality and disability rate around the world, results in apoptosis and necrosis of brain tissue due to ischemia and anoxia (Kang et al., 2003). The pathophysiologic mechanism of ischemic stroke is rather complex, involving excitatory amino acid toxicity, disorders of energy generation, oxidative stress injury, inflammatory reaction and other factors (Abas et al., 2010).

A number of published studies show that orexins, especially OA, play a neuroprotective role in cerebral ischemic injury and ischemia–reperfusion injury (IRI). CSF OA levels undergo persistent declines in patients with cerebral infarction (Nishino and Kanbayashi, 2005). In subarachnoid hemorrhage (SAH) patients, CSF OA levels are low during the 10 days after the precipitating event. In regard to complications of delayed ischemic neuronal deficit (DIND) resulting from symptomatic vasospasm in SAH patients, CSF OA levels are higher in patients who do not develop DIND (Dohi et al., 2005). Consistent with these results, the number of OA-containing neurons is obviously higher on the ischemic than the non-ischemic side (Kitamura et al., 2010). Moreover, administration of OA significantly decreases brain infarct area.

A few studies suggested that OX1R is associated with cerebral ischemic injury. OX1R is highly induced not only in neurons, but also in astrocytes and oligodendrocytes, in rat and mouse models of cerebral ischemia, suggesting that OA and OX1R play important roles in ischemic insult (Nakamachi et al., 2005). In addition, animal models of cerebral ischemia have shown that the expression of OX1R increases in the brain, which was relevant to decreases of OA concentration in cerebrospinal. In particular, Irving et al. (2002) reported that mRNA and protein levels of OX1R, but not OX2R, were elevated in rat ischemic cortex after permanent middle cerebral artery occlusion (MCAO). In that study, CSF OA levels were transiently elevated in comparison with the baseline 24 h after ischemia, gradually decreased on the 2nd and 4th days after ischemia, and finally returned to the baseline level on the 7th day. These changes were correlated with elevated expression of OX1R in the CA1 on the 1st and 2nd days following ischemia. These changes suggest that the dynamics of orexin and OX1R may play functional roles in neuronal damage associated with transient ischemia (Dohi et al., 2006).

Intracerebroventricular injection of OA inhibited nerve injury induced by the MCAO of mice on the 3rd day after ischemia (Harada et al., 2011). In another report, OA significantly ameliorated neurologic deficits and decreased the area of the infarct area in subjects suffering from cerebral IRI. The mechanism underlying the neuroprotective effect of OA is likely related to a reduction in the number of apoptotic cells and activation of HIF-1α. Moreover, treatment with a HIF-1α inhibitor suppresses the stroke-related increase in HIF-1α and reverses the neuroprotective effect of OA (Yuan et al., 2011).

In rat cerebral cortex, orexins markedly increase the survival of neurons in a concentration-dependent manner. This pro-survival ability of orexins is related to a reduction in caspase-3 activity (Sokoowska et al., 2012). Harada et al. (2013) found that OA prevents cerebral ischemic neuronal damage by promoting the expression of brain-derived neurotrophic factor (BDNF). In a MCAO model using orexin/ataxin-3 transgenic mice, OA treatment obviously altered the expression of TNFα and IL-6 at the mRNA level, implying that a chronic inflammatory response is involved in this process (Xiong et al., 2013).

In light of these findings, the orexin/receptor system protects neurons against cerebral ischemia and IRI by regulation of anti-apoptotic and inflammatory responses. A deeper understanding of the signaling pathways underlying orexin/receptor-promoted neuroprotection might facilitate the design of new therapies for cerebral ischemia and IRI.

### Orexin/Receptor Signaling and Addiction

Drug addiction is a chronic and relapsing disorder distinguished by compulsive drug-seeking behavior at the expense of other activities. Early in 1954, Olds and Milner identified the LH as an important brain region in the reward system (Olds and Milner, 1954). Velley et al. (1983) answered the long-standing question of whether the intrinsic neurons in LH are involved in self-stimulation. The orexin/receptor system, especially via OX1R, was reported to be strongly related to addiction to drugs, especially alcohol, nicotine and cocaine (Smith et al., 2009; Dehkordi et al., 2017; Moorman et al., 2017). Although OX2R is generally agreed to be closely associated with arousal and sleep regulation (Willie et al., 2003), orexin/OX2R signaling has also been reported to be an important mediator of drug-seeking behavior in several reports (Smith et al., 2009; Cason et al., 2010; Shoblock et al., 2011).

### Orexin/Receptor Signaling and Ethanol Seeking

Accumulating reports reveal that orexins and their receptors are abnormally expressed in some models of alcohol consumption. For example, a high dose of ethanol can increase the concentration of orexin in the LH (Morganstern et al., 2010). Hypothalamic orexin–containing neurons are significantly more abundant when animals are exposed to ethanol availability (Dayas et al., 2008). Administration of OA into the paraventricular nucleus (PVN) and LH induces ethanol intake in ethanol-drinking rats (Schneider et al., 2007). In accordance with those observations, intraperitoneal administration of the OX1R antagonist SB334867 decreases the ethanol intake and preference of high ethanol–preferring rats (Moorman and Aston-Jones, 2009). In addition, various concentrations of SB334867 (10, 15 and 20 mg/kg) decrease operant self-administration of 10% ethanol (Richards et al., 2008). Another experiment provided proof that SB334867 also significantly decreases the responding and break points for ethanol (Jupp et al., 2011).

Additionally, both voluntary ethanol drinking and intragastrically administered ethanol increase gene expression of OB in the aPVT, suggesting an important role for OB in regulation of ethanol intake (Barson et al., 2015). Furthermore, injection of the OX2R antagonist TCSOX229 into the aPVT may significantly decrease ethanol consumption. Moreover, peripheral injection of JNJ-10397049, another selective OX2R antagonist, decreases ethanol self-administration, behavior related to acquisition, expression, and reinstatement of ethanol conditioned place preference (CPP), and ethanol-induced hyperactivity in mice (Shoblock et al., 2011). Similarly, injection of TCSOX229 into the nucleus accumbens (NAc) decreases self-administration of ethanol. However, TCSOX229 does not alter cue-induced reinstatement of ethanol seeking. Unlike OX1R, OX2R probably plays a more prominent role in ethanol self-administration than in cue-conditioned ethanol seeking (Brown et al., 2013).

**Figure 7:**
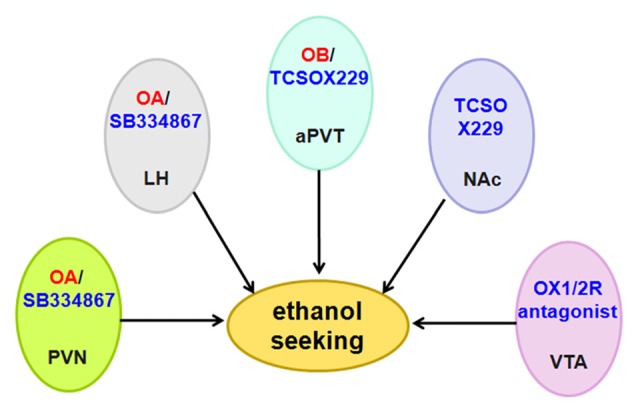
Schematic diagram illustrating the roles of orexin/receptor in ethanol seeking. Administration of OA or OB into the PVN, LH and aPVT induces ethanol seeking. In accordance with these findings, intraperitoneal administration of OX1R or OX2R antagonist decreases ethanol intake and ethanol self-administration. Abbreviation: PVN, paraventricular nucleus; LH, lateral hypothalamus; aPVT, anterior paraventricular thalamus; NAc, nucleus accumbens; VTA, ventral tegmental area.

Anderson et al. (2014) used the OX1R antagonist SB334867, the OX2R antagonist LSN2424100, and the mixed OX1/2R antagonist almorexant (ACT-078573) to evaluate the effect of OX1R and OX2R on ethanol self-administration. The results from different operant experiments indicated that OX1R and OX2R decrease ethanol self-administration, although they have non-specific effects on consummatory behavior (Anderson et al., 2014). Almorexant decreases ethanol self-administration when injected directly into the VTA (Srinivasan et al., 2012; Figure 7).

### Orexin/Receptor Signaling and Nicotine Seeking

The orexin/receptor system is associated with the coordination of physiological and behavioral responses to nicotine treatment. OX1R is activated in rat models of nicotine reinforcement. Furthermore, the OX1R antagonist and the OX2R antagonist both decrease nicotine self-administration in a dose-dependent manner (LeSage et al., 2010). Intracerebroventricular OA reinstates the behavior induced by nicotine seeking, but SB334867 effectively blocks the nicotine motivational response (Plaza-Zabala et al., 2010). Hollander et al. (2008) showed that low doses of SB334867 in the insula effectively decrease nicotine intake in rats, as well as the motivation to obtain the drug. Importantly, SB334867 selectively decreases nicotine intake without altering the response to food. In addition, SB334867 decreases nicotine self-administration (Hollander et al., 2008). In C57BL/6J nicotine-dependent mice, the syndrome of nicotine withdrawal is diminished in SB334867-pretreated and preprohypocretin-knockout mice, but the syndrome was not observed at all in mice pretreated with the OX2R antagonist TCSOX229 (Plaza-Zabala et al., 2012).

Nicotine increases the percentage of Fos-expressing cells in orexin-secreting neurons, and this effect is diminished by nicotinic antagonist (Pasumarthi et al., 2006). Pretreatment with the OX1R antagonist SB334867, but not the OX2R antagonist TCSOX229, decreases reinstatement of nicotine seeking. Moreover, PKC signaling modulates relapses to nicotine-seeking behavior (Plaza-Zabala et al., 2013). GSK1059865, another highly selective OX1R antagonist, significantly decreases voluntary ethanol intake in ethanol-dependent mice, but not in non-dependent mice (Lopez et al., 2016).

Taken together, these data indicate that nicotine-enhanced OA/OX1R transmission plays an important role in regulating the stimulatory properties of nicotine. Therefore, receptor antagonists may be key neurobiological substrates necessary for maintenance of nicotine addiction in human smokers.

### Orexin/Receptor and Cocaine Seeking

The orexin/receptor system was shown to stimulate cocaine intake. OA leads to a dose-dependent reinstatement of cocaine seeking and obviously increases self-stimulation thresholds, indicating that it negatively modulates the activity of the brain reward circuit (Boutrel et al., 2005). Intraperitoneal SB334867 dose-dependently reverses conditioned reinstatement induced by cocaine (Martin-Fardon and Weiss, 2014), and OX1R-knockout mice self-administer far less cocaine than wild-type mice (Hollander et al., 2012). Co-injection of SB334867 with OA cannot block cocaine seeking, whereas another OX2R antagonist, TCSOX229, fully prevents the cocaine seeking induced by OA, indicating that the priming effects of OA injection into the pPVT are mediated by OX2R (Matzeu et al., 2016). Pretreatment with SB334867 significantly attenuates conditioned cocaine seeking elicited by a drug-associated context (Smith et al., 2010). Likewise, SB334867 decreases cocaine self-administration, likely by modulating the mesolimbic DA system (Espana et al., 2010).

Microinjection of SB334867 into central amygdala (CeA) dose-dependently decreases cocaine intake in model rats. Moreover, SB334867 blocks GABAergic neurotransmission within the medial CeA, indicating that GABAergic neurotransmission is involved in the orexin-mediated regulation of cocaine intake (Schmeichel et al., 2017). Microinjection of SB334867 into bilateral VTA, or the AMPA receptor antagonist CNQX, diminishes reinstatement of cocaine seeking, whereas PEPA, a positive modulator of AMPA receptors, completely reinstates the attenuation caused by SB334867. This implies that reinstatement of cocaine-seeking behavior is dependent on interactions between OX1R and AMPA receptors in the VTA (Mahler et al., 2013). Borgland et al. (2006) reported that OA potentiates NMDAR EPSCs in VTA DA neurons via activation of PLC/PKC-dependent signaling pathways. OX1R antagonists block sensitization to cocaine and occlude cocaine-induced potentiation of excitatory currents. Thus, orexin/OX1R signaling in VTA is critical for behavioral sensitization to cocaine (Borgland et al., 2006). In mice harboring a knockout of the orexin prepro-peptide, CPP for cocaine fails to develop, and DA release and uptake are diminished. Further, reduced DA signaling in knockout mice persists following administration of cocaine, suggesting that orexin-mediated regulation of reinforcement might be associated with DA neurotransmission (Shaw et al., 2017). SB334867 decreases the motivation to self-administer cocaine and attenuates cocaine-induced enhancement of DA signaling. Combined with the observation that orexin knockout decreases the DA response to cocaine, these findings suggest that orexin/OX1R modulates cocaine reinforcement, likely through the mesolimbic DA system (Espana et al., 2010).

Blockade of OX1R or simultaneous blockade of OX1R and OX2R diminishes the effects of cocaine on DA signaling and the motivation to take cocaine. By contrast, blockade of OX2R alone has no significant effect on DA signaling or self-administration. These findings suggest differential involvement of the two receptors, with OX1R playing a more important role than OX2R in the regulation of DA signaling and cocaine self-administration (Prince et al., 2015). However, repeated cocaine stimulation may increase OX2R protein expression in the NAc, with no effect on OX1R, OA, or OB in this region. In comparison with NA, OX2R is not altered by cocaine in the frontal cortex, hippocampus, VTA, or dorsal striatum. Remarkably, upregulation of OX2R can persist up to 60 days after discontinuation of cocaine (Zhang et al., 2007).

Together, these findings suggest that orexin/OX1R is necessary for cocaine self-administration, whereas orexin/OX2R is less important. Future studies on the role of the orexin/receptor system in the regulation of cocaine seeking will facilitate the development of interventions against cocaine seeking using antagonists.

## Orexin/Receptor Signaling and Alzheimer’s Disease

AD is a degenerative disease of the central nervous system characterized by progressive cognitive dysfunction and behavioral impairment. The pathogenesis of AD is summarized as follows: extracellular β-amyloid deposition leads to neuronal degeneration; hyperphosphorylation of tau protein results in formation of neurofibrillary tangles, undermining the normal functions of neurons and synapses. Some documents have reported that AD is associated with the loss of orexin-containing neurons and a certain degree of impairment in orexin neurotransmission, indicating that the orexin/receptor system plays an important role in AD pathogenesis. In a study, the number of OA neurons was reduced by 40% in AD patients, and the concentration of OA in CSF was 14% lower (Fronczek et al., 2012). On the contrary, CSF orexin levels are reported to elevate in AD patients (Liguori et al., 2016, 2017). Futhermore, the upregulation of CSF OA in AD is correlated with amyloid-β_42_ levels (Gabelle et al., 2017). These contradictory conclusions indicate that the relationship between orexin/OXR system and AD is complexity. The more extensive and deeper studies on orexin/OXR system in AD are needed.

In amyloid precursor protein (APP)/presenilin 1 (PS1) transgenic mice, knockout of the orexin gene significantly diminishes the degree of Aβ pathology. Inversely, rescue of orexinergic neurons in APP/PS1 increases the amount of Aβ pathology, indicating that orexin modulates Aβ pathology in the brain (Roh et al., 2014). Urrestarazu and Iriarte (2016) speculated that AD patients suffer from some disturbances in the secretion of orexins which brought about sleep disorders, subsequently enhanced amyloid-β level, ultimately contribute to the pathogenesis of the AD.

OA/B treatment impacts the formation of actin filaments around Aβ fibrils and downregulates phagocytosis-regulating molecules such as PI3K, Akt and p38-MAPK, demonstrating that orexin can impair Aβ degradation through the suppression of phagocytosis and autophagic flux (An et al., 2017). Moreover, expression of OXRs and GPR103 is reduced in AD due to Aβ-plaque formation and tau hyperphosphorylation. Furthermore, in an *in vitro* AD model, OXRs and GPR103 form functional heterodimers, exerting a neuroprotective effect by activating ERK_1/2_ signaling pathway.

Considering that orexin overexpression causes Aβ accumulation and tau-mediated neurodegeneration, orexin receptor antagonists represent potential preventive/therapeutic strategies against AD. However, more studies are urgent to clarify the role and mechanism of the orexin/OXR system in the pathophysiology of AD.

## Conclusion and Perspectives

Via the wide projections of orexin-containing neurons, their complicated circuits with other types of neurons and the diffuse distribution of orexin receptors, the orexin/receptor system is involved in the regulation of multiple CNS functions. When the orexin-containing neurons are damaged or lost, they cause imbalances between orexin-containing neurons and the associated neurons. Once these neurotransmitter systems are broken or disrupted, symptoms of neurological disease appear. At present, an effective strategy for neurological disorders related to the orexin/receptor system is to selectively promote the activity of orexin-containing neurons, or instead to block the function of the orexin receptor using a receptor antagonist. In particular, pharmacological interventions targeting orexin receptors have been shown to be effective measures against neurological diseases. However, definitive assignment of physiological roles of the orexin/receptor system in neurological diseases requires in-depth pharmacological and molecular investigations. Given our increasing understanding of the orexin/receptor system, this knowledge will certainly be applied to clinical treatment of neurological disorders in the near future.

## Author Contributions

CW wrote the manuscript and designed the figure. QW proposed suggestions for revision. BJ revised the manuscript. YP assisted in designing the figure. CX helped to arrange references. BC made suggestions for revision. BB provided intellectual thoughts. JC provided intellectual thoughts and proposed supplementary content.

## Conflict of Interest Statement

The authors declare that the research was conducted in the absence of any commercial or financial relationships that could be construed as a potential conflict of interest.
